# Experiences and perceptions of care-seeking for febrile illness among caregivers, pregnant women, and health providers in eight districts of Madagascar

**DOI:** 10.1186/s12936-022-04190-x

**Published:** 2022-07-07

**Authors:** Rachel Favero, Catherine M. Dentinger, Jean Pierre Rakotovao, Laurent Kapesa, Haja Andriamiharisoa, Laura C. Steinhardt, Bakoly Randrianarisoa, Reena Sethi, Patricia Gomez, Jocelyn Razafindrakoto, Eliane Razafimandimby, Ralaivaomisa Andrianandraina, Mauricette Nambinisoa Andriamananjara, Aimée Ravaoarinosy, Sedera Aurélien Mioramalala, Barbara Rawlins

**Affiliations:** 1 Maternal and Child Survival Program, 1776 Massachusetts Ave, NW, Suite 300, Washington, DC 20036 USA; 2 Malaria Branch, Division of Parasitic Diseases and Malaria, Center for Global Health, Centers for Disease Control and Prevention, Atlanta, GA USA; 3 US President’s Malaria Initiative, US Centers for Disease Control and Prevention, Antananarivo, Madagascar; 4 Maternal and Child Survival Programme, Antanaimena Immeuble Santa, Lot II 3ème étage 101, Antananarivo, Madagascar; 5 National Malaria Control Programme, Ministry of Health, Antananarivo, Madagascar

**Keywords:** Care-seeking, Malaria, Community, Madagascar, Febrile illness, Pregnant women

## Abstract

**Background:**

Prompt diagnosis and treatment of malaria contributes to reduced morbidity, particularly among children and pregnant women; however, in Madagascar, care-seeking for febrile illness is often delayed. To describe factors influencing decisions for prompt care-seeking among caregivers of children aged < 15 years and pregnant women, a mixed-methods assessment was conducted with providers (HP), community health volunteers (CHV) and community members.

**Methods:**

One health district from each of eight malaria-endemic zones of Madagascar were purposefully selected based on reported higher malaria transmission. Within districts, one urban and one rural community were randomly selected for participation. In-depth interviews (IDI) and focus group discussions (FGD) were conducted with caregivers, pregnant women, CHVs and HPs in these 16 communities to describe practices and, for HPs, system characteristics that support or inhibit care-seeking. Knowledge tests on malaria case management guidelines were administered to HPs, and logistics management systems were reviewed.

**Results:**

Participants from eight rural and eight urban communities included 31 HPs from 10 public and 8 private Health Facilities (HF), five CHVs, 102 caregivers and 90 pregnant women. All participants in FGDs and IDIs reported that care-seeking for fever is frequently delayed until the ill person does not respond to home treatment or symptoms become more severe. Key care-seeking determinants for caregivers and pregnant women included cost, travel time and distance, and perception that the quality of care in HFs was poor. HPs felt that lack of commodities and heavy workloads hindered their ability to provide quality malaria care services. Malaria commodities were generally more available in public versus private HFs. CHVs were generally not consulted for malaria care and had limited commodities.

**Conclusions:**

Reducing cost and travel time to care and improving the quality of care may increase prompt care-seeking among vulnerable populations experiencing febrile illness. For patients, perceptions and quality of care could be improved with more reliable supplies, extended HF operating hours and staffing, supportive demeanors of HPs and seeking care with CHVs. For providers, malaria services could be improved by increasing the reliability of supply chains and providing additional staffing. CHVs may be an under-utilized resource for sick children.

## Background

Malaria remains a public health and socio-economic problem in Madagascar and has been increasing in some areas of the country [1, 2]. Despite evidence that prompt diagnosis and treatment of malaria reduces severe morbidity and mortality, [3] care is often delayed or not pursued at all in Madagascar [4], The Malaria Indicator Survey (MIS) showed that only 40% of children with a fever in the last 2 weeks sought care in the formal health system [4]. This may be in part because approximately 60% of the population lives farther than 5km from a health facility (HF) [5] and has limited access to transportation.

To improve access to care, Madagascar engages community health volunteers (CHV), an unpaid cadre of workers with limited training who deliver basic services including malaria diagnosis and treatment to children less than 5 years of age (CU5), select family planning services, and health promotion activities in their communities [6]. These services are packaged as integrated community case management (iCCM), a strategy has been shown to improve care-seeking behavior by community residents for iCCM illnesses, including malaria. [7] An estimated 36,000 CHVs cover the country of Madagascar, with two CHVs designated per fokontany (village), the smallest administrative unit. They are supported by one of the country’s 2200 HFs in 1549 communes within the 114 health districts across the country [8]. HFs, in contrast, provide basic health services to community residents including malaria care for all ages, and serve as referral centres for CHVs [9]. However, HFs frequently have shortages of clinical staff, equipment, and commodities (e.g., rapid diagnostic tests [RDT] and anti-malarial medications) [10]. Most HFs in Madagascar are public, but an estimated 496 private HFs also serve residents throughout the country. Small-scale studies in Madagascar have shown that delays in care-seeking for suspected malaria are influenced by cost (real or perceived), perceptions of disease severity, travel time/distance from HFs, and the quality of care expected to be received [11, 12]. Poor quality of malaria care has been documented in low- and middle- income (LMIC) countries, with one study finding that 35% of children under 5 across 25 LMICs received under or over treatment for malaria [13]. Another study found that community members did not perceive malaria to be a serious health problem and thus were not likely to seek care promptly [14]; and another study found that taboos, including seeking care at western-style facilities, can also affect care-seeking [15]. In many Malagasy communities, residents seek care in the informal health sector (e.g., relatives, traditional healers, small businesses selling medicine and health care products). Reported reasons for preferring the informal sector include convenience, availability of drugs even in isolated villages and during off-hours, social and geographic proximity to vendors, and affordability (lower cost and/or payment on credit) [14, 16]. Self-medication early in the course of illness is also common [17].

In 2005, Avedis Donabedian developed a quality-of-care framework that includes three components as measurements for improvement. These components include structure measures, process measures and outcome measures [18]. This assessment sought to explore and document structure related elements through questions to participants about the attributes or detriments of seeking care in the formal healthcare sector at facilities and with CHVs. Questions to healthcare providers and CHVs about what would better support their use of national malaria treatment guidelines, including availability of commodities and workload, were also employed to understand structural barriers to quality care. Process measure elements were documented through questions to pregnant women and caregivers on their perceived quality of care and wait times in the formal sector. Knowledge tests and in-depth interviews (IDI) with health providers and CHVs sought to document provider understanding of correct malaria case management guidelines. Improvements, where needed, in these structure and process measures will support advances in outcomes to include reduced patient wait times, improved patient experience and improved health outcomes [18].

Comprehensive studies to describe care-seeking for febrile illness among vulnerable populations (i.e., children and pregnant women) in Madagascar are limited. Thus, a survey that includes perspectives of caregivers, pregnant women, and providers to describe the determinants of care-seeking for febrile illness among caregivers of children < 15 years of age (caregivers) and pregnant women across Madagascar was conducted.

## Methods

### Key definitions

The study team created a list of key definitions to standardize language used in the assessment protocol, tools and final dissemination products. Having an agreed upon nomenclature for study elements helped ensured consistency and quality throughout the assessment. For a list of key definitions, see Table 1.

**Table 1:** Key definitions, Care-Seeking Behavior Study, Madagascar, 2018

Term	Definition
*Caregive*r	An individual ≥ 18 years of age who provides care for a child < 15 years of age
*Community health volunteers (CHV)*	Unpaid health personnel with limited training who deliver basic health care services in their communities
*Community residents (CR)*	Caregivers or pregnant women who participated in the study
*Formal health system*	Government-recognized HF (called *Centre de Santé de Base* [CSB] in Madagascar) and CHVs
*Health facility (HF)*	A clinic or health center directly supported by the Government of Madagascar (public) or by private entities (private)
*Health Provider (HP)*	A physician, nurse or midwife who provides clinical services in public or private HF
*Informal health system*	Relatives, traditional healers, businesses that sell medications and/or health care products, pharmacies
*Non-user of services (non-user)*	Caregivers or pregnant women reporting febrile illness during the previous two weeks who did not seek care from the formal health system (regardless of care-seeking from the informal health system)
*Participants*	Any study participant including CRs, HPs, and CHVs
*Pregnant woman*	A female who reports she is pregnant
*Rural commune*	The smallest administrative unit located in a rural area (eg, more than 15 km from the district capital) in Madagascar as defined by the national statistical offices
*Urban commune*	The smallest administrative unit located in an urban area (eg, less than 15 km from the district capital) in Madagascar as defined by the national statistical offices
*Users of health services (user)*	Caregivers or pregnant women who sought care for febrile illness from the formal health system

### Study setting and sample

**Fig. 1:**
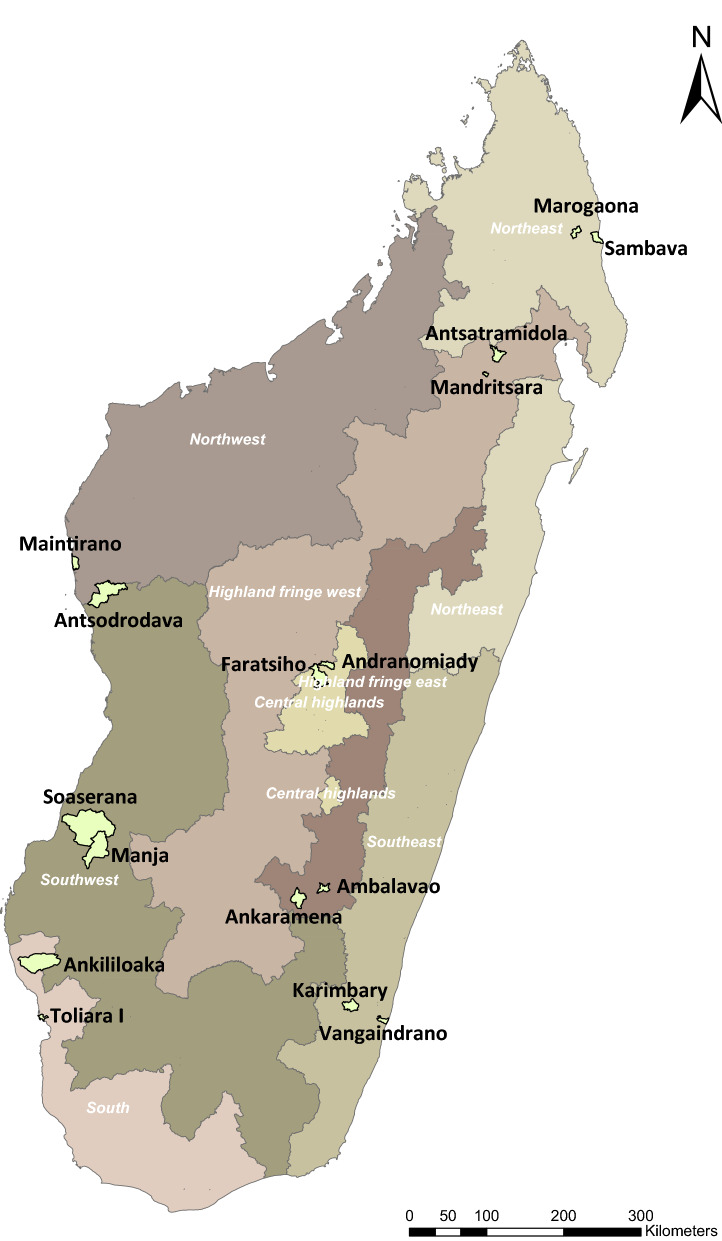
Study Commune Boundaries, Care-Seeking Behavior Study, Madagascar, 2018

Madagascar is divided into eight malaria ‘operational ecozones’ based on *Plasmodium falciparum* prevalence (*Pf*PR) during 2010–2015 [19]; one health district from each of these zones was included in the study. In each zone, health districts, which are comprised of several health communes, were purposefully selected based on higher malaria incidence relative to other districts in the zone according to routine health information data. Two communes (administrative unit below district) per district, one urban and one greater than 15 km from the capital of the district, were randomly selected for inclusion (Table 2, Fig. 1). The one lower-level public HF (CSB I or CSB II) associated with each of the communes was selected for inclusion. One private HF (CSB-equivalent) associated with the commune, if available, was also selected at random.

**Table 2:** Malaria operational zones, Districts and Communes, Care -seeking Behavior Study, Madagascar, 2018

Malaria operational zone	District	Urban commune	Rural commune
Central highlands	Faratsiho	Chef Lieu* Faratsiho	Andranomiady
Highland fringe west	Mandritsara	Chef lieu Mandritsara	Antsatramidola
Highland fringe east	Ambalavao	Chef lieu Ambalavao	Ankaramena
South	Tulear II	Chef lieu Tulear II	Ankililoaka
Northeast	Sambava	Chef lieu Sambava	Marogaona
Southwest	Manja	Chef lieu Manja	Soaseranana
Northwest	Maintirano	Chef lieu Maintirano	Antsondrondava
Southeast	Vangaindrano	Chef lieu Vagaindrano	Karimbary

^*^ “Chef Lieu” in French translates to “Main town” in English

### Study participants

#### HPs and CHVs

All consenting HPs and affiliated CHVs from study HFs who had been working in the HF for at least six months were invited to participate in IDIs. In Madagascar, most CSBs only employ one HP who supports all client services. In facilities where more than one provider was available, HPs who support antenatal care (ANC) services and provide services to CU5 were asked to participate in the assessment.

#### Caregivers and pregnant women

Consenting caregivers and pregnant women were eligible if they resided within 10 km of the HF. These participants, called community residents (CR), were recruited for IDIs, and focus group discussions (FGD) by CHVs. For IDIs, one CR who was a health care user and one who was a non-user were selected and for FGDs, eight CR (four users and four non-users) per commune were selected. CRs for FGD could be either caregivers or pregnant women.

### In-depth interviews and focus group discussions

For IDIs, at least one participant from each rural and each urban fokontany was selected from each of the following categories: public HP, private HP, CHV, and either a caregiver/pregnant woman user or non-user of services. Interviewers used semi-structured questionnaires to understand who/what influences timely care-seeking. Among HPs and CHVs, interviews were designed to understand providers’ treatment practices for patients with febrile illness, and to identify barriers and facilitators to their adherence to national febrile illness case management guidelines.

For FGDs, four participants from each rural and each urban fokontany were selected from each of the following categories: caregiver/pregnant woman who was a user of services and caregiver/pregnant woman who was a non-user of services. Users of services were randomly selected from facility registers and given the option to participate. Non-users of services were identified with the help of CHVs in the fokontany. FGDs were used to explore perceptions of quality of care in the health system. Health system users and non-users were combined to promote interaction and understand care-seeking motivations and expectations; however, their responses to questions were tracked according to whether they identified as a user or non-user.

### Provider knowledge tests

Knowledge tests regarding managing and treating febrile illness among children and pregnant women were administered to providers. The tests included both open-ended and multiple-choice questions; respondents could give more than one response. Knowledge tests were administered to a random selection of 15 of the participating HPs.

### Commodity assessments

Inventory and pricing data from the national Logistics Management Information System (LMIS) on malaria commodities for the previous six months were abstracted from registries at public and private HFs and from CHV records.

### Data collection

Data collectors received 1 week of training on study protocols and standard operating procedures. Study tools were pre-tested at HFs near Antananarivo, the country’s capital, and updated based on pre-test findings. After approval from local authorities, data collection took place during October 2017. IDIs lasted for 45 min and FGDs for about one hour. During FGDs, one study team member facilitated while another took notes. Rules of saturation [20] applied whereby data collectors discontinued interviews or FGDs once common themes were documented. Knowledge tests were administered to providers over 30 min. Data abstraction from LMIS took about one hour per facility.

### Data analysis

#### Qualitative

Before IDI and FGD data collection began, an initial codebook was developed based on assessment themes and study objectives. The codebook was designed to highlight differences and recurrences of themes that emerged throughout discussions. The study team translated the IDI and FGD transcripts from Malagasy to French and met regularly to standardize coding implementation through checks on coding consistency, discuss emerging codes and update the codebook. Microsoft Excel 2016 (Microsoft Corporation, Redmond, WA) was used to perform data analysis. Study team members coded participant responses using the established codebook and then quantified the coded responses in Excel. Emerging themes and specific quotes were used to illustrate the findings.

#### Quantitative

Provider knowledge tests were scored to determine the percentage of correct responses. Key variables for LMIS data included percent of facilities that had (a) at least one malaria RDT in stock, (b) a stock-out of malaria RDTs for more than one day in the previous 6 months, (c) at least one artemisinin-based combination therapy (ACT) course (for any age) in stock and (d) a stock-out of an ACT (for any age) for more than one day in the last six months. Additional information was collected on the price charged to patients for malaria RDTs and ACT in HFs and with CHVs.

### Ethical considerations

The protocol was determined to be non-human subjects research by the Malagasy Ethics Committee, the Johns Hopkins University School of Public Health Institutional Review Board and by the US Centers for Disease Control and Prevention (CDC). Ethical guidelines applicable to human subjects’ research were nonetheless followed during data collection. Written informed consent was obtained from all participants.

## Results

The assessment was conducted from September to December, 2017. A total of 83 IDIs, 16 FGDs, 24 LMIS reviews and 15 HP knowledge tests were completed (Table 3).

**Table 3:** Health facilities and participants, by data collection method, Care-Seeking Behavior, Madagascar, 2018

	Logistics management information system register review	In-depth Interviews	Focus Group Discussions (FGD)	Provider knowledge test
*Subject category*				
Public health facility	10	16	–	8
Private health facility	8	15	–	7
Community HEALTH volunteer	6	5	–	–
User of services- Caregivers of children under 15 and pregnant women	–	27 users (14 caregivers and 13 pregnant women)	8 FGD (80 users [43 caregivers and 38 pregnant women])	–
Non-user of services- community residents (CR- non-user): Both caregivers of children under 15 and pregnant women	–	20 non-users (11 caregivers and 9 pregnant women)	8 FGD (64 non-users [34 caregivers and 30 pregnant women])	–
*Total*	24	83	16 FGD, 144 Participants	15

^*^FGDs included both users and non-users of services together in the same FGD

### Perception of fever and malaria among community residents

When asked what diseases could cause a fever, malaria was the first illness cited by all CRs. CRs said that malaria could cause severe illness and lead to death, and that in pregnant women, malaria could cause harm to the fetus including low birth weight, birth defects and death. The most cited causes of malaria included mosquitoes, climate change, soil, lack of personal hygiene and consumption of raw foods. According to some non-users, malaria could be caused by use of cold water that has been heated by the sun (CR-non-user, pregnant woman, rural commune, Faratsiho), sorcery (CR-non-user, caregiver, urban commune, Sambava), transmission by an infected person (CR-non-user, pregnant woman, rural commune, Mandritsara) or eating fruit that has been bitten by mosquitoes (CR-non-user, urban commune, caregiver, Tulear II).

*“[Malaria] can be caused by foods we have consumed. For example, we eat mangos and mosquitoes lay eggs in mangos.” (CR-non-user, caregiver, urban commune, Tulear II)*

Most participants said that bed nets were the best tool to protect against malaria. Almost all participants were aware that fevers might need treatment and most stated they would seek treatment of some kind.

### Community resident approach to care-seeking for febrile illness

Almost all CRs indicated that they would first treat a fever at home with herbal remedies, paracetamol, liquids, and rest. This was true for both pregnant women and caregivers. The primary reason for not seeking immediate care at a HF was lack of finances for travel and consultation fees. If home treatment did not result in reducing the fever, CRs from urban areas were likely to seek care in pharmacies, hospitals, or private clinics, while those in rural areas were likely to visit a public HF or traditional healers. Very few CRs, urban and rural, mentioned seeking care from CHVs as an initial step. Reported care-seeking delays ranged from one day to 1 week after fever onset.

*"I first give my child a massage, then I make him drink herbal tea …. or I make him egg yolk with lemon and honey. Then I fumigate and give him paracetamol and cotrimoxazole [an antibiotic]. If none of this works, then I take him to the hospital or the health centre." (CR-user, caregiver, rural commune, Sambava).*

### Perceptions of the formal health system among community residents

CRs felt that private-sector HPs were more welcoming, had shorter wait times and provided better care than public facility HPs but that pharmaceuticals and consultations were more expensive. CRs expressed an inability to pay for malaria commodities, travel, and food for HF visits.

*"We do not have money, so buying medicine [at facilities] is difficult. 200 Ariary, 300 Ariary, (about $0.07), where do you want me to find this money? We do not even have anything to eat so where are we going to find money for drugs?" (CR non-user, caregiver, rural commune, Tulear II).*

While a few caregivers said that they experienced respectful care at public HFs, most caregivers and all pregnant women disclosed that they frequently felt bullied or disrespected by public HF staff. They also noted long wait times and lack of clinical staff at public HFs and felt that traveling to a HF could be a waste of time and money.

*"There are times when…there is no one to provide care at the [HF]. We do not know how many days we must wait: 3, 4 and even 5 days and everything is still closed. After you have to go to [another facility] and pay a lot of money for the trip." (CR-user, rural commune, Sambava).*

### "The doctor is always late. There are many people waiting. You arrive at 7 or 8 am, but you will not be seen until 11 am." (CR non-user, rural commune, Faratsiho).

CHVs were perceived as being socially and geographically closer to the people they serve, mixing with the communities in their daily lives and making it easier for them to share advice and recommendations with CRs. However, CHVs were not considered by the majority of CRs to be healthcare providers but rather as distributors of mosquito nets, vitamins, and family planning products. CHVs were recognized as outreach and advisory workers with good advocacy skills. In the context of malaria, CRs said that CHVs are those who make available malaria RDTs for CU5.

*"We do not see the reason to go to CHVs; let them do outreach. People do not believe in their treatment capacity. On the other hand, they do sell drugs and RDTs." (CR-user, rural commune, Sambava).*

### Perceptions of traditional healers among community residents

CRs consulted traditional healers due to fears, taboos, discrimination, and costs associated with the formal health system. Several CRs said they prefer traditional healers because they only gave homeopathic or natural remedies.

### “I eat leaves and then I recover.” (CR-nonuser, pregnant woman, rural commune, Faratsiho)

Respondents also noted that some CRs believe that traditional healers are the only ones that can heal afflictions caused by poisonings or spirits and mentioned that some conditions that cannot be cured in hospitals.

*"Especially when the patient has a high temperature and there are frequent convulsions, people think that there is a spirit and that it is only the traditional healer who can remove it.” (HP, rural commune, Vangaindrano).*

Table 4 summarizes key perceptions of CRs regarding types of care facilities and providers.

**Table 4:** Community Resident perceptions of private and public health providers in health facilities, community health volunteers and traditional healers, Care-Seeking Behavior Study, Madagascar, 2018

Type of care	Community resident perceptions (Positive)	Community resident perceptions (Negative)
Public health facility/provider	•Public HPs are experienced and can provide care in case of severe diseases •Bednets are distributed free of charge ••Consultations are free	•Drugs can be expensive •Drugs, equipment, and commodities are not well managed and are frequently out of stock •Long wait times •Sometimes the number of providers is insufficient, and providers are only available in the morning •Unsatisfactory reception of patients by clinical and administrative staff, poor treatment of patients by trainees, lack of listening capacity, lack of respect for patients •Lack of confidentiality •Sometimes the facilities are poorly maintained
Private HF/provider	•Health providers are nicer and more welcoming [than public providers] •There is little or no waiting time •Case management is of good quality and there is frequent follow up •Clinical examinations are properly performed •Pharmaceuticals are of good quality •In general, quality of care is better [than public facilities]	•There are no private facilities in some communes •Consultations and pharmaceuticals are more expensive [than public health facilities]
Community health volunteer	•CHVs are more welcoming than clinical staff in HFs •Patients do not have to pay very much to see CHVs •Drugs are cheaper [than they are at health facilities] or free •CHVs are accessible •Community residents are used to seeing CHVs in their community	•CHVs do not generally provide care, but are more focused on giving advice and recommendations, especially for children under 5 years of age •Frequent stockouts of malaria commodities including RDTs •CHVs do not have a lot of training
Traditional healers	•Their services cost less compared with services in the formal health system •Some CRs trust them because they treat diseases with massage and natural remedies •Proximity of traditional healers due to communities' geographical remoteness and distance from HFs •Some CRs visit them because they fear being referred to a hospital •Patients feel welcomed •Very little waiting time •Habits and customs: It is taboo to see a doctor and receive shots •Belief that there are diseases that hospitals cannot cure •The need to possess "ody" (natural medicines to cure ailments) when people lack information and awareness and tend to view diseases as evil •It is felt that formal healthcare providers sometimes discriminate against certain groups of people and traditional healers do not	•The advice is not accepted by the medical community within facilities •Some CRs regard individuals who use traditional providers as ‘seekers of witchcraft’ •Some CRs go out of habit, not necessarily because it is the best choice for care •The term ‘country people’ is given to some CRs who visit traditional care providers, implying that they are uneducated or have outdated ways of behaving

### Costs of febrile illness and malaria case management for community residents

CRs reported lack of finances as a primary barrier to seeking care within 24 h of fever onset. Table 5 details care costs by facility and provider type. Private HFs charge for consultations, malaria RDTs and some pharmaceuticals; these services and commodities are typically free in public HFs or with CHVs.

**Table 5:** Costs (US dollars) associated with malaria case management, by type of healthcare provider, Car-Seeking Behavior Study, Madagascar, 2018

Cost element	Public HF	Private HF	Community health volunteers
Consultation for a CU5	Free	$0.55—$2.77	Free
Consultation for a pregnant woman	Free	$0.28—$0.55	-
Malaria RDTs	Free	$0.03—$0.28	$0.03 – $0.14
Artesunate + amodiaquine (AS/AQ, which is an ACT)	Free	Free*	$0.01 – $0.03
Injectable quinine	$0.08	$0.19—$0.55	-
Quinine tablets	$0.03	Free	N/A
Injectable artesunate	$0.02	$0.04 – $0.29	-

Note: As of 5 October 2019, 1 USD = 3,608 Malagasy Ariary (MGA)

Data Source: Facility LMIS (logistics management information systems)

^*^Respondents noted that AS/AQ was free to them. Private suppliers sometimes receive commodities free of charge from international implementing partners with the understanding that they will deliver them free of charge to clients

CR-users noted that distance, commodity stock-outs, poor quality of care, and absence of providers were the main barriers to care-seeking. CR non-users were more likely to note fears of contracting other illnesses, beliefs that diseases were caused by evil, and lack of habit of going to HFs (Table 6).

**Table 6:** Barriers to community residents seeking care for febrile illness in the formal healthcare system, by user/non-user status and healthcare provider/community health volunteer, Care-Seeking Behavior Study, Madagascar 2018

Barriers according to CR-user respondents	Barriers according to CR non-user respondents	Barriers according to healthcare providers and CHVs
•High cost of care in hospitals and private facilities •Distance from health facilities •Lack of qualified personnel at public health facilities •Accessibility of drugs on the market/self-medication •Frequent absences of workers from health facilities •Stockouts of suitable drugs in both HFs and with CHVs	•Lack of financial means •Distance from health facilities •Afraid to go to hospitals •Fear of contracting another illness caused by taking drugs •Use of traditional healers •Cultural beliefs according to which some diseases are caused by evil and cannot be cured through the formal health system •Accessibility of drugs on the market/self-medication •Lack of habit of going to health facilities	•Local culture still encouraging use of traditional healers •Lack of habit of going to health facilities among some community residents •Accessibility of drugs on the market/self-medication •Distance from health facilities •Fear of dying in the hospital. People tend to wait until illnesses are serious which leads to higher numbers of deaths in hospitals

CRs and HPs had similar suggestions for improving care-seeking including HFs being more geographically accessible to CRs, cultivating understanding of the importance of prompt care-seeking, improving commodity availability, and creating a more welcoming environment (Table 7).

**Table 7:** Health provider, community health vounteers and community resident suggestions for overcoming care-seeking barriers, Care-Seeking Behavior Study, Madagascar, 2018

Community residents	HPs and CHVs
•Improve the geographical accessibility of public health facilities •Strengthen communications and awareness amongst CR for malaria prevention and timely care-seeking •Ensure availability of malaria related commodities at health facilities •Upgrade existing hospitals (materials, logistics); •Strengthen human resources at HFs (in terms of number and skills) •Ensure more professionalism and welcoming behavior from healthcare providers •Work to reduce insecurity and bandits (i.e., "dahalo") •Reduce fees charged to patients at HFs •Improve HF-patient communication	•Improve the geographical accessibility of public HFs by building more HFs •Strengthen communications and awareness of CRs for malaria prevention and timely care-seeking •Ensure availability of malaria-related commodities at health facilities •Improve patient welcome and reception •Strengthen the capacity of CHVs and other healthcare providers •Provide incentives to CRs to seek care in HFs

### Availability of malaria commodities at HFs

All age-formulations of artesunate-amodiaquine (AS/AQ), the first-line anti-malarial recommended by the National Malaria Control Programme (NMCP), were available in 8 of the 10 public facilities surveyed; availability of almost every essential malaria product was lower in the private facilities. Availability of AS/AQ formulations for use by CHVs to treat infants and CU5 was 33% and 17%, respectively. RDTs were available in 9 of the 10 public HFs but in only four (50%) private HFs. During the previous 6 months, RDT stock-outs of at least 1 day occurred in 4 (40%) public HFs and in 75% (6 of 8) private HFs; two private HFs had had at least 90 days of stock-outs, one public HF had a stock-out of at least 60 days and one for at least 30 days. Four of six (67%) CHVs had an RDT on the day of the survey (Table 8).

**Table 8:** Availability and stockouts of malaria RDTs and antimalarials by type of provider, Care-Seeking Behavior Study, Madagascar, 2018

	Public health facilities (n = 10)		Private health facilities (n = 8)		Community health volunteers^***^ (n = 6)	
	Availability* (%)	Stock-outs** (%)	Availability (%)	Stock-outs (%)	Availability	Stock-outs
RDT	90	40	50	75	67%	50%
AS/AQ for adolescents-adults (14 + years)	80	30	63	50	NA	NA
AS/AQ for young children (1–5 years)	70	60	38	75	33%	83%
AS/AQ for children (6–13 years)	80	40	38	75	33%	83%
AS/AQ for infants (< 1 year)	70	40	25	87	17%	83%
Injectable quinine	40	70	50	50	NA	NA
Quinine tablet	40	80	25	75	NA	NA
Injectable artesunate	40	70	13	87	NA	NA

Source: Logistics management information systems (LMIS)

*NA* Not applicable

^*^Availability is defined by presence of at least one of the specified products on the day of the assessment

^**^Stock-outs include facilities that have encountered stock-outs for at least one day of a product in the last 6 months prior to the assessment

^***^Policies at the time of the study did not allow CHVs to treat children < 5 years of age

Delivery delays and stock-outs at suppliers are the main causes of stock-outs mentioned by HPs and CHVs (Table 9). Respondents also report that stock-outs are due to failure to send orders, underestimation when quantifying needs, and suppliers not filling orders completely. According to one CHV, an increase in cases of fever can also lead to stock-outs.

**Table 9:** Reported* causes of stock-outs of RDTs and antimalarials, by type of facility or community health volunteer, Madagascar Care-Seeking Behavior Study, 2018

	Public health facilities (n = 10) (%)	Private health facilities (n = 8) (%)	Community health volunteers (n = 6) (%)
Late delivery by supplier	50	25	33
Stock-outs at suppliers	20	13	33
No order sent	30	0	17
Underestimation by facility when quantifying needs	10	0	17
Supplier does not deliver according to order	10	0	17
Increase in malaria cases	0	0	17

^*^Responses recorded from HF personnel who reviewed LMIS with study team

### Provider knowledge tests

Of 15 HPs, 13 (87%) scored 85% or higher on the knowledge test. All HPs indicated that a patient should have a positive diagnostic test for malaria before receiving malaria treatment. All providers also spontaneously answered that a child with a positive RDT should receive ACT. When asked what treatment a woman who is four months pregnant should receive, providers indicated the following treatments: ACT (11 [73%]) (the correct answer), SP (one), quinine (one) and injectable artesunate (two).

### Health providers and national malaria case management guidelines

HPs generally agreed that they do their best to follow the national malaria treatment guidelines, even though copies of the guidelines are largely absent from HFs. Providers felt that covering many responsibilities at their facilities sometimes hindered their ability to provide quality care. Providers also said that they are sometimes unable to follow guidelines due to drug stock-outs in which case they use available formulations and adjust the dose according to the patient’s weight.

*"For [a patient who is] 1 to 5 years, half of the dosage for 6 to 13 years is used. For [a patient who is] 6 to 13 years, half of the adult dosage is taken. For patients over 14, a calculation is also done in case we do not have [their correct formulation]." (HP, rural commune, Manja).*

## Discussion

This is the first large scale assessment to document the care-seeking practices of both caregivers for children under 15 years of age and pregnant women in Madagascar that describes the underlying determinants for those practices. The study includes perceptions of the quality of care for febrile illness and malaria within and outside the formal health sector among these vulnerable populations. The study also includes perspectives from those who use the system and those who do not, along with healthcare providers’ perspectives on care-seeking practices of their patients, perceptions of the quality of care within their facilities, and their malaria care knowledge. Among CRs, distance to health facilities, costs of transportation and services, concerns about facility closures and commodity stock outs, perceptions of poor care quality, and cultural beliefs are common reasons for delayed care-seeking. Among providers, who generally scored well on knowledge tests, commodity stock outs were identified as barriers to providing quality malaria care.

### CR and HP perceptions of malaria and where to get care, ‘perception barriers’

CRs recognized the linkage between febrile illness and malaria and were aware that malaria is a serious disease. This may have improved since a 2014 study in Madagascar, [21] which found that CRs did not perceive malaria to a be serious or worrisome health problem. Increases in community messaging supported by the NMCP and implementing partners over the past five years [21, 22] may have contributed to these improvements. However, some CRs, particularly those who were ‘non-users’ of the formal health care system, ascribed the cause of malaria as spiritual or sorcery and noted that doctors are not the correct provider to treat such conditions. More outreach and communication are needed regarding the causes of malaria and the services provided by health facilities and traditional healers. Additionally, some non-users of services mentioned that they were not in the habit of seeking care at HFs; as such, they do not think to attend. Messaging around care-seeking for fever at health facilities might also work to habituate community residents to HF use. Being habituated to seeking care from a particular HF, CHV or traditional provider emerged from study data as a motivator for where one seeks care. Identifying a place as ‘usual’ or ‘common’ in your or your community’s experience has been shown in other studies to be a primary motivator for seeking care at that location [23].

### Structural barriers

Structural barriers including cost, distance to health facility, staff shortages and stock outs of essential supplies emerged as challenges to care-seeking for febrile illness. A perception of high costs associated with obtaining HF services was pervasive across CR respondents, both pregnant women and caregivers, even for public health facilities. CRs’ reported inability to pay even 200 MGA (equivalent to $0.05 USD) for malaria medication illuminates the lack of accessibility to healthcare in many parts of Madagascar. Malaria-related care, including the clinic visit and medication, are officially free in public facilities in Madagascar; and clients being charged suggests inappropriate application of official policy. However, travel costs, lost productivity and inconvenience, which were also identified as prohibitive, are not covered, and must be carefully weighed given that CRs might arrive to find unstaffed or unstocked facilities. HPs also reported that cost and travel time were barriers to CRs seeking prompt care. CRs perceived that the quality of care was better in private facilities. This seemed to result from shorter wait times and more welcoming attitudes; however, CRs noted these facilities tended to be more expensive, perhaps limiting their use. Removing fees in the formal health care sector could increase service use [24, 25]. This was shown in Madagascar according to a 2014 study which found that when fee exemptions were introduced, service provision increased for all patients including CU5 and maternity consultations [26].

Surprisingly, CHVs were not perceived as routine providers for CU5 despite being identified as having RDTs and ACT. The most recent Malaria Indicator Survey supports this sentiment, finding that only 7% of children who sought care for a fever did so with a CHV [4]. Methods to correct this perception could include communication strategies so that CRs are aware of CHV capabilities and that RDTs and ACT are elements of proper malaria care. Improving the actual and perceived quality of care offered by CHVs, in addition to improving commodity availability, may also contribute to an increase in timely care-seeking behaviour for fever, as has been documented in past studies across LMICs [7].

All respondents identified staff shortages in HFs as an ongoing problem. For CRs, this translated to long wait times or HF closures, both identified as reasons they delayed care-seeking. For HPs, this resulted in excess work. In Madagascar, HPs are often alone at post in rural settings and there is no coverage during illnesses, trainings, meetings, or annual leave so these result in HF closure. CRs also noted unwelcoming behavior including bullying on the part of HPs. This HP behavior might result in part from being overworked, having limited support and managerial oversight, and lack of commodities and supplies to properly care for their patients [24]. Updates to pre-service curricula and in-service policies and practices could help to improve respectful care practices. The use of CHVs for the care of CRs necessitates a thorough review of the same challenges faced by HFs, including improving CHV training and supervision, assuring reliable stocks of essential materials, and addressing CHV workloads. Other countries have successfully implemented quality outreach services, as is the case in Bangladesh with their door-step model [27]. Investing in compensation for CHVs would need to be considered for a similar implementation in Madagascar.

CRs and HPs identified that stock outs were problematic. For CRs, stock outs contributed to the perception that HFs provided poor care quality, which was not worth seeking. HPs noted that stock outs inhibit their ability to provide quality services. The lack of malaria RDTs and medications hinders not only care-seeking among CR but also perceived efficacy of HPs to deliver quality care [10].

Notably, the themes that emerged from the data regarding care-seeking and quality of care were advanced by both pregnant women and caregivers. This may reflect the fact that many of the challenges that Malagasy communities are encountering are rudimentary, health system related, and thus become difficult for all users, regardless of age, gender, or pregnancy status.

Study participants’ recommendations to facilitate prompt care-seeking included decreasing HF wait times, assuring adequate supplies, and addressing HF staff shortages and behavior. Providing quality in-service training to HPs on respectful care has been shown to improve the reception patients receive and the level of support and communication they feel throughout consultations at HFs [28, 29]. Simultaneously delivering this training and supervision to HPs as part of in-service curriculum in Madagascar on malaria relevant topics could also improve quality of febrile illness and malaria care in health facilities in Madagascar. Instituting incentives to HPs and making these benefits contingent on maintaining regular open hours within their facility may reduce the risk to patients that facilities will be unattended when they arrive. Offering larger incentives to HPs in Madagascar who are willing to work in extremely remote areas may also advance services in those areas. Increasing the number of trained clinical and nonclinical staff in HFs in Madagascar can also help to avoid large HP workload and reduce wait times for CRs. Finally, developing and streamlining an efficient commodities management system along with staff training and supervision may help maintain consistent stocks of commodities. Building the capacity of all stock managers on a regular basis through training and monthly reviews in both the private sector and facility managers in each health district should be prioritized. Regular quality checks of commodity management tools and reporting can also help to sustain reliable malaria and febrile illness related stocks.

## Limitations

Participant demographics were not documented in this study; therefore, findings cannot be described by gender, age, marital or socioeconomic status, which might have provided additional insights. However, the CRs were sampled from different malaria zones and community sizes, and several key findings were common across these diverse populations, suggesting the findings are likely common across different demographic groups. In addition, the generalizability of the provider knowledge tests is limited as the sample size was small and the participants not randomly selected; however, it was reassuring that HPs performed well suggesting knowledge deficits were not a driver of poor-quality malaria services. Finally, though hospitals were not included in the sampled facilities, some respondents reported going to hospitals to receive care. As such, respondent perspectives may have included their thoughts on hospital services even though they were specifically asked about primary care facilities.

## Conclusions

Timely care-seeking for febrile illness to prevent malaria morbidity in Madagascar remains a challenge even though community residents recognize that malaria is a serious disease. Several structural barriers to care-seeking, including high travel and opportunity costs and long distances to HFs, emerged as important barriers. Additionally, frequent stock-outs of essential malaria testing and treatment supplies were noted by both community members and providers as a critical reason why some caregivers and pregnant women perceived that care was not worth seeking. Addressing these barriers may result in more timely care-seeking from community residents in Madagascar.

## Data Availability

The datasets generated and/or analysed during the current study are not publicly available because they contain personally identifiable information but are available from the corresponding author on reasonable request. Data would be de-identified before sharing.
